# Dynamic Changes in Endophytic Microorganisms and Metabolites During Natural Drying of Licorice

**DOI:** 10.3389/fmicb.2021.740721

**Published:** 2021-10-14

**Authors:** Ting Li, Guangxi Ren, Dan Jiang, Chunsheng Liu

**Affiliations:** School of Chinese Pharmacy, Beijing University of Chinese Medicine, Beijing, China

**Keywords:** natural drying, water change, metabolism, endophytic microorganisms, licorice

## Abstract

The method of drying licorice is an important factor affecting the quality of the final product. To determine the best processing method of licorice postharvest, we investigated the interaction of increasing aridity between the endophytic microorganisms and the accumulation of metabolites. Samples from the roots of licorice growing along an aridity gradient during the natural drying process were collected, and the metabolic components, the content of the main active substances and the dynamic changes of the endophytic microbial community were assessed. The glycyrrhizic acid and liquiritin contents decreased slightly or remained flat during natural drying, whereas those of liquiritigenin and isoliquiritigenin increased slightly. Moreover, the Shannon index of endophytic microbial diversity of licorice was the highest in the fresh period and showed a downward trend during the drying process. When the licorice were fresh, Cladosporiaceae and Burkholderiaceae were the dominant family present, but after drying, Nectriaceae and Enterobacteriaceae were the dominant families. A similar trend was also found in which the differential metabolites of licorice were reduced during natural drying. Furthermore, correlation analysis between dominant families and differential metabolites showed that there was a correlation between the two. Therefore, fresh processing is an effective drying method to ensure the quality of licorice. This study revealed the relationship of endophytic microbiota and changes in the licorice metabolites during different stages of drying, which provided a scientific basis for the drying method of licorice.

## Introduction

*Glycyrrhiza uralensis* Fisch. (Licorice) is the most commonly used bulk medicinal material in traditional Chinese medicine. Licorice and its extracts have anti-inflammatory and antiviral properties (Chen et al., 2021; van de Sand et al., 2021). It is also an important additive in cosmetics, healthcare products, tobacco and other industries, and its annual demand is huge (Hayashi and Sudo, 2009; Shakeri et al., 2018; Yin et al., 2020). The licorice plant needs to be processed in a series of steps to become a medicinal material. Drying licorice is the most critical step in this process, ensuring the licorice does not rot due to high water content (Icier et al., 2021). Recent studies have focused on the microwave drying and vacuum freeze drying of licorice (Balbay and Sahin, 2012; Li et al., 2017). However, in general, sun-drying is the method primarily used in cultivation areas; that is, licorice is dug out once it has grown for approximately 3years, gathered into small bundles in the cultivation field, dried in the sun, remoisturized with water and finally cut into decoction pieces. However, this particular method of drying licorice in the sun takes a significant amount of time, which can negatively affect the quality and effectiveness of the final medicinal materials (Icier et al., 2021). Therefore, the analysis of the content of the effective components of licorice in the sun-drying process would be helpful in characterizing the best drying and processing technology of licorice production areas, helping to improve the quality of licorice.

Endophytic microorganisms live in various tissues and organs of healthy host plants without negatively affecting the plant’s health or functions (Wu et al., 2021). Endophytic microorganisms have important effects on plant growth, development and the accumulation of metabolites (Lugtenberg et al., 2016; Zhou et al., 2020; Liu and Wei, 2021; Santos et al., 2021; Singh et al., 2021). It has been reported that Bacillus pumilus can alleviate the drought stress of licorice and increase the accumulation of licorice metabolites (Xie et al., 2019). Endophytic microorganisms are affected by a variety of external factors, including changes in the water content (Huang et al., 2021; Tian et al., 2021). As an important part of licorice, the microbial community composition and function will also change as the water content changes during the drying process. However, the effect of licorice on the functional microbial community during the natural drying process is still largely unknown. Therefore, a clearer understanding of the composition and function of the microbial community during the drying process provides a scientific basis for perfecting licorice production, which is conducive to perfecting the production area and processing of licorice, and provides a scientific basis for obtaining high-quality licorice.

In recent years, researchers have begun using metabolomics to study the types and quantities of plant endogenous metabolites and their changes under the action of internal and external factors. Metabolomics has unique advantages in uncovering these patterns, such as high throughput sequencing, rapid separation, high sensitivity, and widespread use (Jiang et al., 2021; Li et al., 2021). The metabolic components of traditional Chinese medicines are relatively complicated, and the existing detection and analysis methods have been mostly used to focus on one or several known components, making it difficult to achieve a comprehensive quality analysis. Therefore, metabolomics technology can provide an effective method for the qualitative and quantitative analysis of traditional Chinese medicine. At present, metabolomics technology has begun to be used to study the synthesis and transformation of metabolites during the growth and storage of Chinese medicinal materials (Ma et al., 2021). For this reason, metabolomics was used to analyze the dynamic changes in the drying process of licorice in this study.

Here, we analyzed the changes in the content of the effective components of licorice during the drying process and used the Illumina MiSeq high-throughput sequencing platform to analyze the structure and function of endophytic microorganisms in the licorice during the drying process. At the same time, nontargeted metabolomic sequencing was performed on licorice with different water contents, and the relationship between the changes in metabolites and endophytic microorganisms was studied. In summary, our research provides the diversity and composition of the endophytic microbial community of licorice and the simultaneous changes in licorice metabolites during the natural drying process. In addition, it provides a favorable scientific basis for choosing the best licorice processing method.

## Materials and Methods

### Materials

Wild licorice (all *G. uralensis* Fisch.) was collected from an arid hillside in Suide County, Shaanxi Province, transported on ice and stored at −80°C. It was identified by Professor Chunsheng Liu of Beijing University of Chinese Medicine. Numbers W-1, W-2, and W-3 were three groups of licorice with a thickness of 0.8, 0.9, and 1.1cm, and each group was composed of five licorice roots of the same thickness. The samples were all mixed with five licorice roots to form one sample. The reference substances liquiritin (Lot number: Z13J11X108109), glycyrrhizic acid (Lot number: P24J10F91300), isoliquiritigenin (Lot number: C03A8Q41092), liquiritigenin (Lot number: Z20J8X40265), and glycyrrhetinic acid (Lot number: T27J7X18416) were purchased from Shanghai Yuanye Bio Technology Co., Ltd. (Shanghai, China).

### Drying Method

The fresh licorice root was dried in a simulated production area (natural drying). During the process, the water content was measured every 6days (0day: fresh, 6days: early stage of drying, 12days: middle stage of drying, 18days: completely dried). Then, the previous operation was repeated until the water content of licorice was below 10%, when the medicinal material was considered to have reached the dry standard. At the same time, the licorice root was sampled every 6days (The samples were mixed with five licorice roots to form one sample). The metabolites of the samples were analyzed and determined by HPLC–MS. The licorice was remoisturized and sliced within half a year after it was completely dried, which was also in line with the processing method of licorice. After that, the content of the active ingredients of licorice was tested.

### Extraction and UPLC Analysis of the Effective Components of Licorice

The licorice powder was extracted with 70% methanol and detected by UPLC. Liquiritin, liquiritigenin, isoliquiritigenin, glycyrrhizic acid, and glycyrrhetinic acid were taken in appropriate amounts to prepare mother liquor with a liquiritin concentration of 0.139mg/ml, liquiritigenin concentration of 0.051mg/ml, isoliquiritigenin concentration of 0.065mg/ml, glycyrrhizic acid concentration of 0.24mg/ml and glycyrrhetinic acid concentration of 0.099mg/ml. Then, 0.5, 1, 1.5, 2, and 2.5ml was taken and diluted to 10ml with methanol to a series of concentrations. The chromatographic conditions were as follows: BEH C18 column (100mm×2.1mm, 1.7μm, Waters, United States) acetonitrile (A)-0.1% phosphoric acid water (B); gradient elution: 1min, 10% A; 7min, 14% A; 14min, 21% A; 21min, 30% A; 25min, 45% A; 28min, 70% A; 34min, 10% A; 35min, 10% A. The flow rate was 0.4ml/min, and the injection volume was 1μl. Each experiment was repeated five times.

### Extraction of Metabolites and HPLC–MS Analysis Conditions

One hundred milligram of licorice tissue was added to 800μl of 80% cold methanol solution, which was then crushed by a high-throughput tissue breaker under low temperature conditions. After being mixed, it was placed on ice and ultrasonically extracted for 10min, repeated three times. The sample was placed at −20°C for 30min and centrifuged at 13,000*g* at 4°C for 15min. After centrifugation, the supernatant was collected for detection by LC–MS. The chromatographic conditions were as follows: the column was a BEHC18 column (100mm×2.1mm, 1.7μm, Waters, United States). The mobile phase was acetonitrile (A)-0.1% phosphoric acid water (B); gradient elution: 0min, 95% B; 3min, 80% B; 9min, 5% B; 13.0min, 5% B; 13.1min, 95% B; 16min, 95% B. The flow rate was 0.4ml/min, the column temperature was 40°C, and the injection volume was 10μl. Using positive and negative ion scanning modes, the positive electrode ionization voltage was 3.5kV, the negative electrode ionization voltage was 2.8kV, the ion source heating temperature was 400°C, the sheath gas flow rate was 40psi and the aus gas flow rate was 10psi. Each experiment was repeated six times.

### DNA Extraction and Diversity Sequencing of Endophytic Microorganisms

The licorice root was sampled every 6days for sequencing after sterilization. The sterilization method was as follows: rinsing with purified water for 30min, rinsing with sterile water 2–3 times, disinfecting with 75% ethanol for 2min, rinsing with sterile water 2–3 times, sterilizing with 0.1% mercury for 5min, and rinsing with sterile water 2–3 times. The last rinse was used to verify that it was cleaned. Total DNA was extracted from the samples (licorice roots at 0, 6, 12, and 18days of drying) with a DNA kit (Fast DNA Soil, MP, United Sates) according to the instructions. Primers were designed according to the 16S rRNA and ITS conserved regions. The sequences were as follows: 799F: AACMGGATTAGATACCCKG; 1193R: ACGTCATCCCCACCTTCC; ITS1F: CTTGGTCATTTAGAGGAAGTAA; ITS2R: GCTGCGTTCTTCATCGATGC. The DNA was amplified by PCR, purified and quantified, after which the sequencing library was constructed. Sequencing was performed using Illumina HiSeq 2500. The PE reads obtained by MiSeq sequencing were first spliced according to the overlap relationship, and the sequence quality was controlled and filtered at the same time. OTU clustering was performed after distinguishing the samples. Species annotation and abundance analysis were carried out to reveal the composition of the samples. Alpha and *β* diversity of species was analyzed. In addition, significant species difference analysis was used to explore the differences of samples under different drying times. Picrust2 was used to predict the function of endophytic microorganisms.

### Data Analysis

The content of effective components is expressed as mean±SD. Statistical significance was evaluated using a one-way ANOVA for multiple comparisons (SAS 9.4 Software). A value of *p*<0.05 was considered statistically significant. The raw metabolic data were imported into the metabolomics processing Progenesis QI (Waters Corporation, Milford, United States) for baseline filtering, peak identification, integration, retention time correction, peak alignment, and finally a data matrix of retention time, mass-to-charge ratio and peak area was obtained. Then, the data were preprocessed, and the MS and MS/MS mass spectra information were matched with the metabolic database. The main database used were http://www.hmdb.ca; http://metlin.scripps.edu and other public databases. Correlations between the microbial community and metabolites were carried out based on Spearman correlation coefficients. All sequences produced from Illumina sequencing were uploaded to the sequence read archive (SRA) of the NCBI database. The accession number of all samples is PRJNA748524.

## Results

### Dynamic Changes in Effective Components in Licorice During Drying

**Figure 1:**
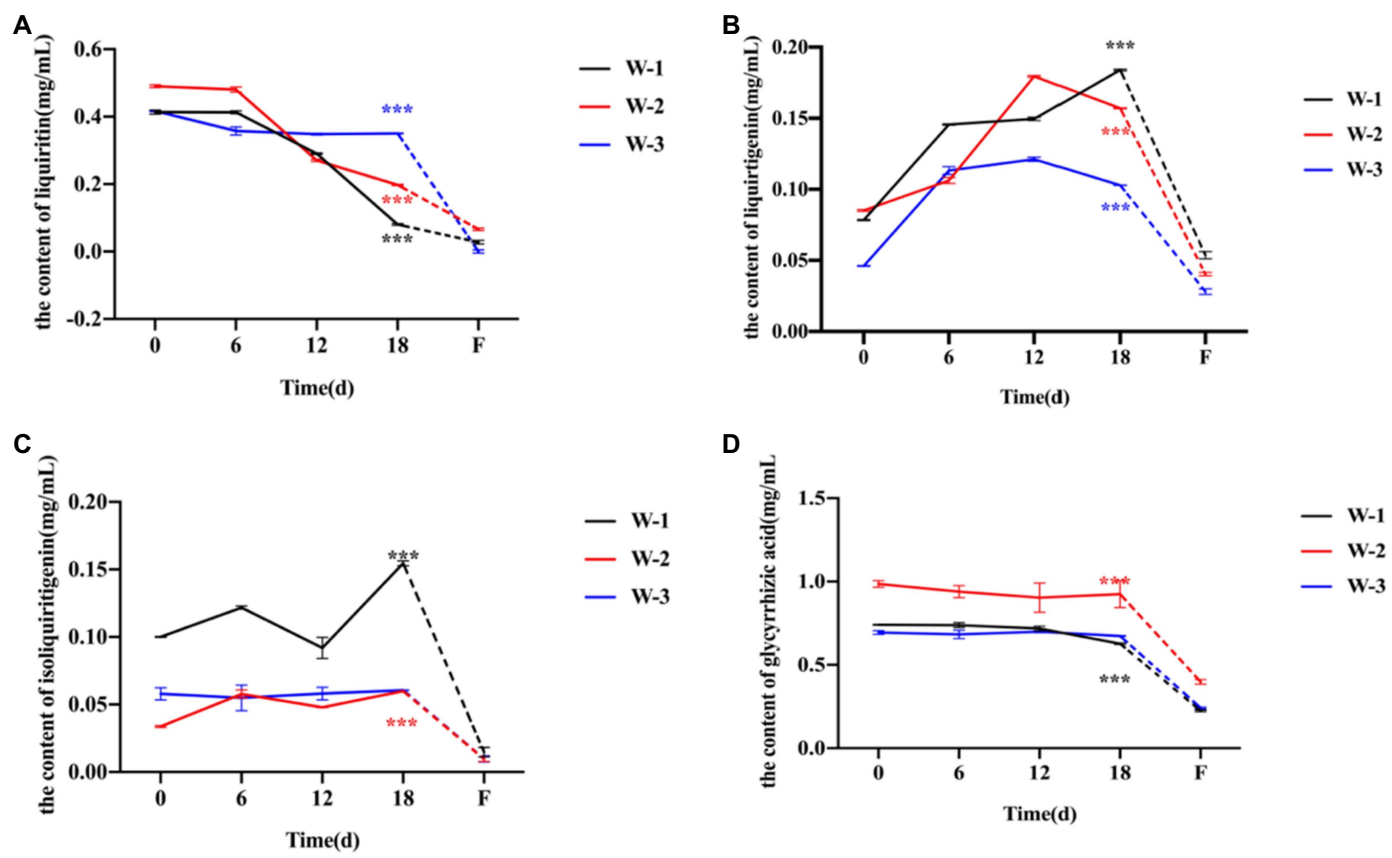
Dynamic changes in the active ingredient content of licorice every 6days during the natural drying process. **(A)** Dynamic changes in liquiritin content during different drying periods. **(B)** Dynamic changes in liquiritigenin content during different drying periods. **(C)** Dynamic changes in isoliquiritigenin content during different drying periods. **(D)** Dynamic changes in glycyrrhizic acid content during different drying periods. The dotted line indicates that the licorice is moistened again after drying for a long time, and *** of different colors indicates a significant difference between fresh and completely dried components of different groups, *p*<0.01. Each sample was made by mixing five licorice roots, and the measurement was repeated five times.

Because the age of wild licorice could not be ascertained, the licorice was divided into three groups according to root thickness, and each group was composed of five licorice roots of the same thickness to eliminate the risk of error caused by unequal root thickness. Five index components were selected for the determination: liquiritin, liquiritigenin, isoliquiritigenin, glycyrrhizic acid, and glycyrrhetinic acid. However, it may have been impossible to detect glycyrrhetinic acid in some samples due to the extremely small concentration in licorice roots, so we did not analyze the glycyrrhetinic acid content of the samples. Compared with fresh licorice, the contents of liquiritin and glycyrrhizic acid in the three groups of samples decreased slightly or remained flat with increasing drying time (Figures 1A,D), while the contents of liquiritigenin and isoliquiritigenin increased slightly (Figures 1B,C). It was speculated that the aglycone was removed from glucose and converted into glycosides or other compounds during the natural drying of licorice. Except for the difference between the content of isoliquiritigenin and that of completely dried isoliquiritigenin in the W-3 group, the contents of liquiritin, liquiritigenin, isoliquiritigenin and glycyrrhizic acid in the other groups were significantly different between fresh and completely dried samples. In addition, after a long period of drying and then moistening with water, the content of all active ingredients in licorice was extremely reduced. Therefore, it was speculated that a large portion of the effective ingredients of licorice will be lost after traditional processing technology (after remoistening and cutting).

### Dynamic Changes in Endophytic Microorganisms During Drying

#### Structural Characteristics of the Endophytic Microorganism Community

**Figure 2:**
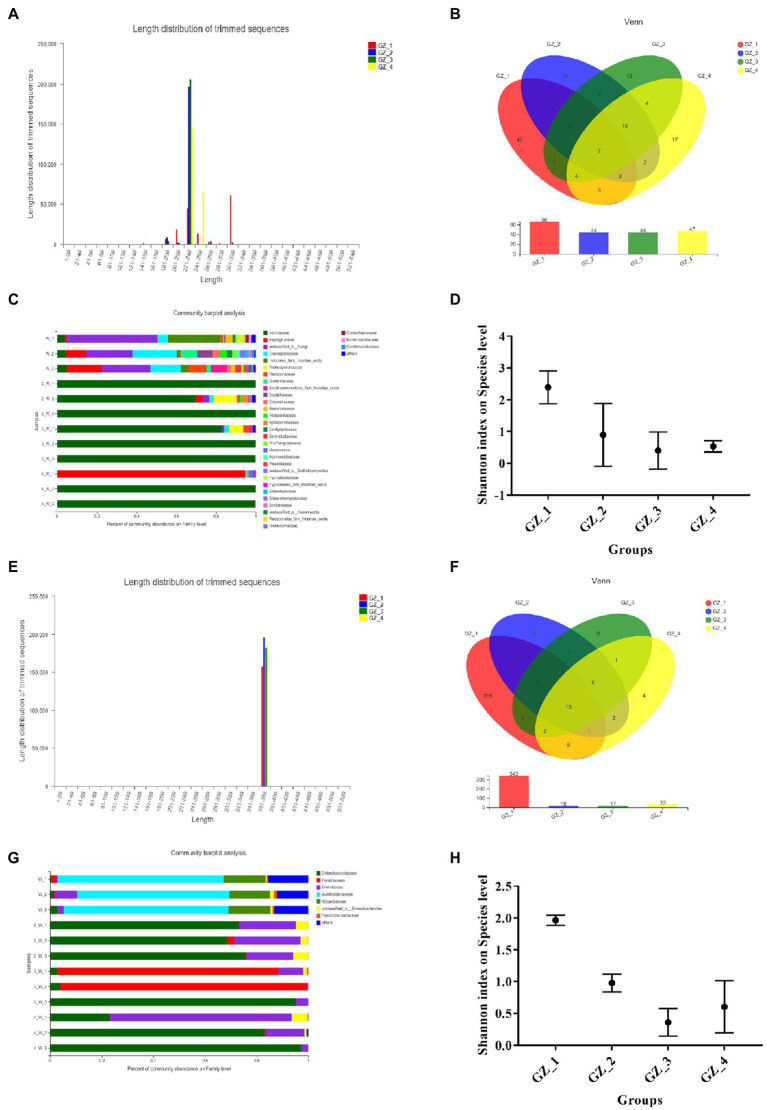
Structural characteristics of the endophytic microbial community of licorice during the natural drying process (GZ_1: fresh licorice; GZ_2: early drying: 6days after drying; GZ_3: mid-drying: 12days after drying; and GZ_4: complete drying: 18days after drying. Each sample was made by mixing five licorice roots). **(A)** The distribution range of the sequence length of endophytic fungi. **(B)** Venn diagram of endophytic fungi in different groups. **(C)** Endophytic fungal community composition. **(D)** Shannon index at the level of endophytic fungal species in different groups. **(E)** The distribution range of the length of the endogenetic bacteria sequencing sequence. **(F)** Venn diagram of endophytic bacteria in different groups. **(G)** Endophytic bacterial community composition. **(H)** Shannon index at the level of different groups of endophytic bacteria.

To better understand changes in microbial communities during drying, 16S rRNA and ITS gene amplification sequences were used to investigate the microbial community within licorice. The analysis results showed that the sequence length of the endophytic fungi was between 180 and 320 bp (Figure 2A), belonging to 128 OTUs (indicating 128 different endophytic fungi), and 66 OTUs were distributed when licorice was fresh, 42 of which were unique. There were 44 OTUs at the beginning of drying, 14 of which were unique. There were 44 OTUs in the mid-drying period, 12 of which were unique. There were 47 OTUs when the licorice was completely dried, 17 of which were unique (Figure 2B). Each experimental group had a different proportion of endophytic fungi. The number first declined rapidly and then remained almost unchanged. At the family level of fungi, unclassified fungi (average abundance of 31.07%), Cladosporiaceae (average abundance of 14.37%), Helotiales-fam-incertae-sedis (average abundance of 10.4%) and Aspergillaceae (average abundance of 9.59%) composed the entire fresh licorice bacterial community. However, Nectriaceae emerged as the dominant family, and the abundance increased dramatically in the slow drying (Figure 2C). The Shannon index was used to estimate the diversity of the microbial community. The Shannon index was 2.39 when the licorice was fresh, and the diversity of endophytic fungi was the highest at this time. In the subsequent drying process, the diversity of endophytic fungi showed a decreasing trend (Figure 2D).

The sequences of endophytic bacteria ranged from 360 to 400bp (Figure 2E), belonging to 350 OTUs. When licorice was fresh, there were 343 OTUs and 315 unique OTUs (Figure 2F). There were 18 OTUs and 17 OTUs in the early and mid-drying stages, respectively, and there were no unique OTUs. When licorice was completely dried, there were 30 OTUs and four of which were unique. At the family level (Figure 2G), Burkholderiaceae (average abundance of 62.25%) and Nocardiaceae (average abundance of 16.19%) composed the entire fresh licorice bacterial community. In the early stage of drying, Enterobacteriaceae (average abundance of 72.52%) and Erwiniaceae (average abundance of 21.83%) were the main dominant bacteria. After the mid-drying period, Yersiniaceae was the main dominant bacteria, with an average abundance of 90.63%. When the licorice was completely dried, Enterobacteriaceae (average abundance of 67.85%) and Erwiniaceae (average abundance of 29.25%) were the main dominant bacteria. When the licorice was fresh, its Shannon index was the highest, indicating that the microorganisms were diverse and abundant at this time (Figure 2H). In short, endophytic fungi or bacteria had the highest diversity in fresh licorice.

#### Analysis of the Microbial Function of Licorice During Slow Drying

Based on ITS and 16S rRNA sequences, PICRUSt2 was used to predict and analyze the function of fungal and bacterial communities in licorice during the drying process. The main types and gene functions of the obtained samples were analyzed to predict their molecular functions to further elucidate the metabolic activity of endophytic bacteria during the drying process (Tian et al., 2021). The MetaCyc pathway results of endophytic fungi showed that the main function of endophytic fungi with the highest abundance was aerobic respiration I and II, palmitate biosynthesis I, glyoxylate cycle, fatty acid and beta, oxidation and GDP-mannose biosynthesis (Supplementary Material 1). Among them, glucose and glucose-1-phosphate degradation also had certain functions. Surprisingly, we unearthed the squalene synthase and glucose transferase (Supplementary Material 2) that encode the triterpene biosynthetic pathway in the KEGG library of endophytic fungi (Kaewkla and Franco, 2021).

**Figure 3:**
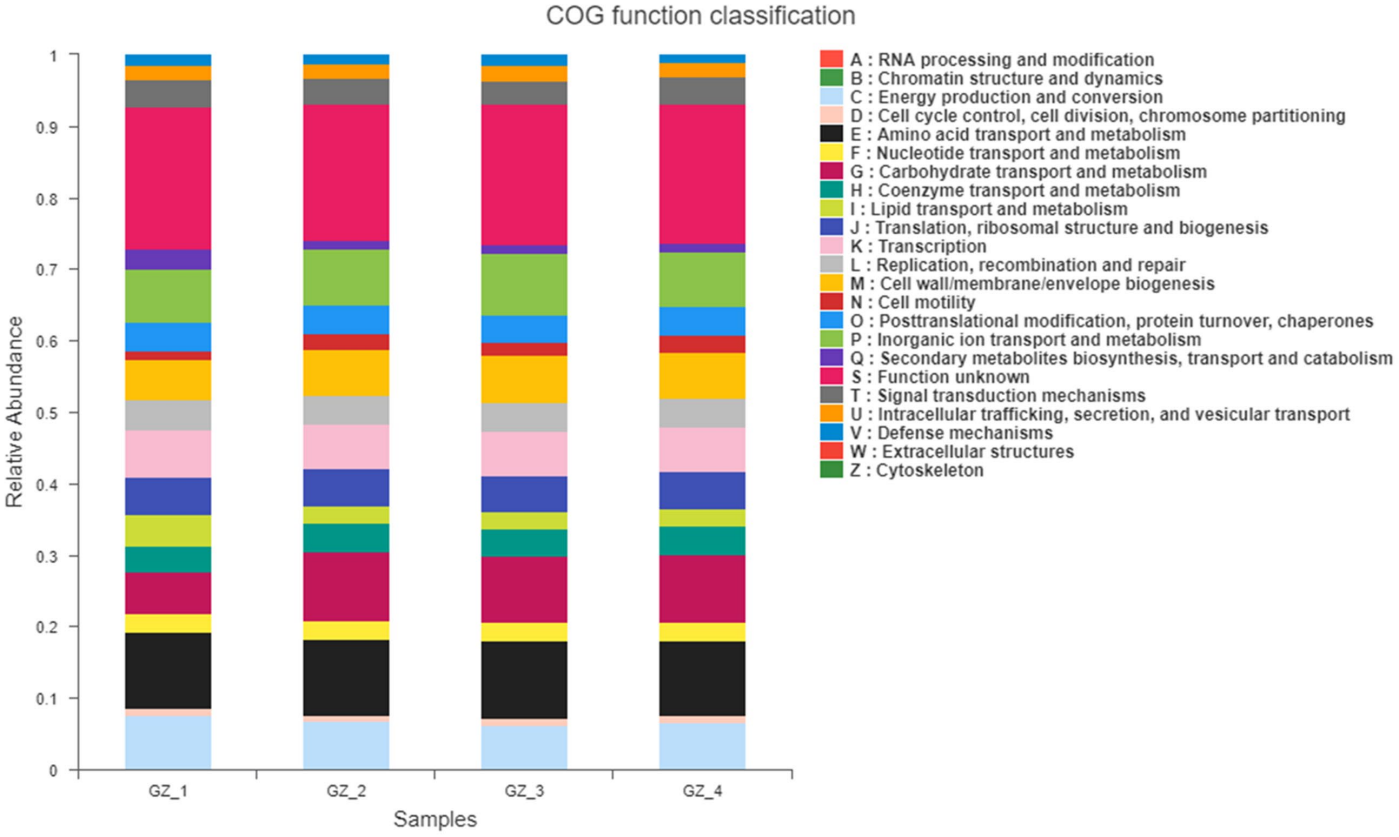
COG function classification statistical histogram (The *x*-axis is different groups. GZ_1: fresh licorice; GZ_2: early drying-6days after drying; GZ_3: mid-drying-12days after drying; and GZ_4: completely drying-after 18days. The *y*-axis is the relative abundance of function).

The MetaCyc pathway results of endophytic bacteria showed that the main function with the highest abundance was pyruvate fermentation into isobutanol (engineering), pentose phosphate pathway (nonoxidative branch), fatty acid β-oxidation I, D-fructose ester degradation, glycine biosynthesis (anaerobic), L-alanine biosynthesis super pathway and the fatty acid biosynthesis initiation super pathway (*Escherichia coli*, etc.). COG functional classification (Figure 3) showed that in addition to the relatively high abundance of unknown functions, cell cycle control, cell division, chromosome partitioning, energy production and conversion, carbohydrate transport and metabolism and organic ion transport and metabolism were still the most important functions.

### Dynamic Changes of Metabolites During Drying

**Figure 4:**
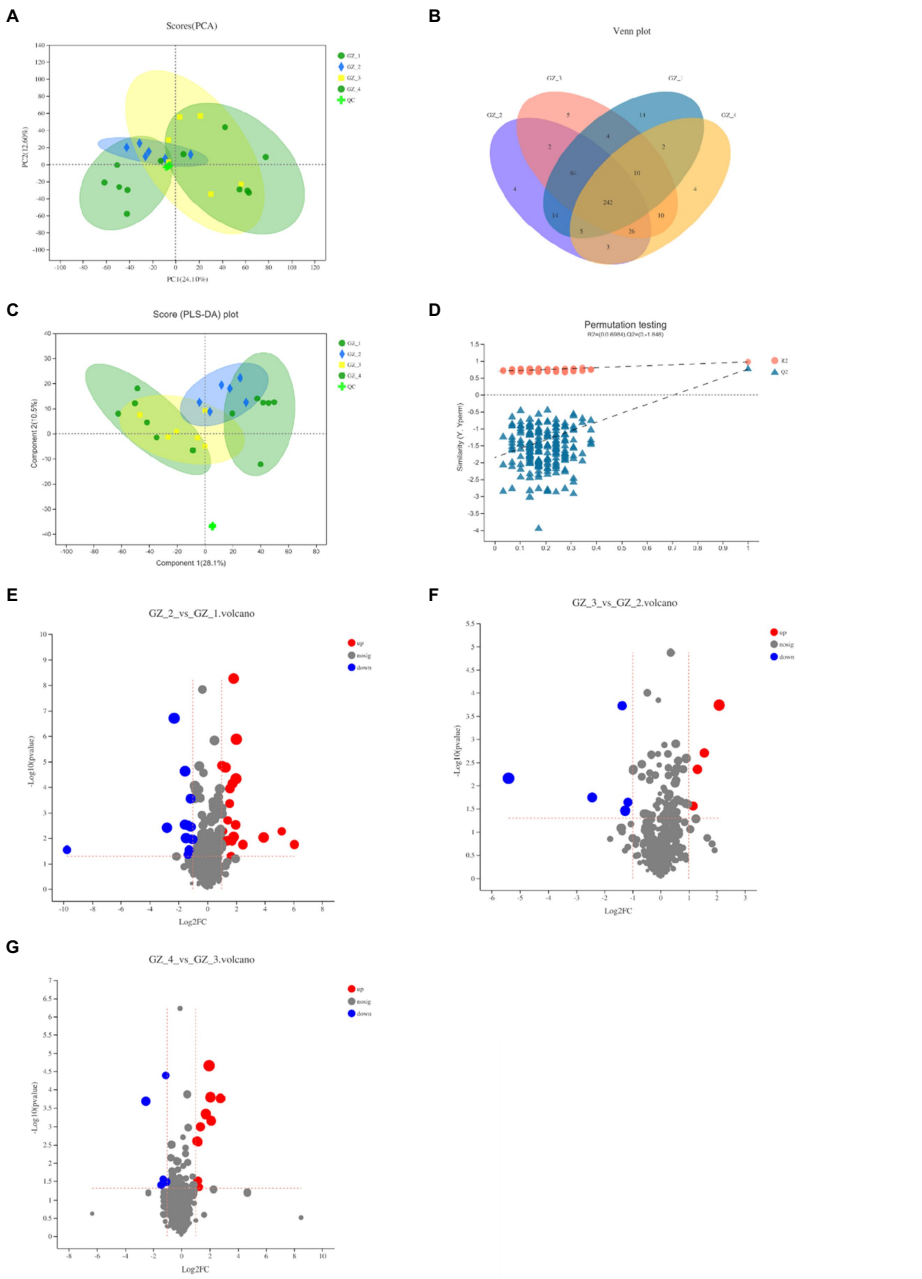
Changes of metabolites of licorice during the four periods of natural drying process (GZ_1: fresh licorice; GZ_2: early drying-6days after drying; GZ_3: mid-drying-12days after drying; and GZ_4: completely dried-18days after drying. Each sample was made by mixing five licorice roots, and the measurement was repeated six times). **(A)** Different periods of cationic PCA scores. **(B)** Venn diagram of metabolites in different periods. **(C)** PLS-DA score chart of different periods. **(D)** PLS-DA model verification. **(E)** Fresh licorice and early drying licorice metabolites’ volcano graph. **(F)** Volcano map of different metabolites of licorice in the early and mid-drying period. **(G)** Volcano map of the different metabolites of licorice in the mid-drying period and completely dry.

The positive and negative ion patterns of the samples were identified, and the structure of the metabolites in licorice was determined by matching the retention time, molecular mass, secondary fragmentation mass spectrum, collision energy and other information. A total of 328 compounds were identified in positive ion mode, and a total of 288 compounds were identified in negative ion mode. Principal component analysis (PCA) was used to comprehensively analyze the clustering trend. The results showed that the metabolite expression patterns of licorice samples were similar during the drying process, and only fresh licorice metabolites were significantly different from other groups. To verify the reliability of the data, QC samples were selected to verify the chromatography and quality inspection system. The PCA score chart showed that the QC samples were close to the origin of the meta-axis and tightly clustered, which showed the stability and reproducibility of the method (Figure 4A). To better reflect the differences in samples during the drying process, a Venn diagram was established for the detected cations. The results showed that the samples contained 14 unique metabolites when fresh. However, licorice contained 4, 5, and 4 unique compounds in the early, middle and complete drying stages, respectively (Figure 4B). In addition, the PLS-DA analysis was established, and the cross-validation of the 200 displacement tests showed that the intercepts R2 and Q2 were 0.6984 and −1.848, respectively (Figure 4D), indicating that the PLS-DA model was reliable. Fresh licorice still had a good degree of separation from the other groups, and the other groups were relatively close, showing no significant difference (Figure 4C).

To further screen the marker compounds related to each group, a volcano map was drawn according to the factor of difference (FC) and value of *p* to analyze the differences between the fresh licorice and the early drying period, the early drying period and the mid-drying period, and the mid-drying period and the complete drying. When fresh licorice was slowly dried for 6days, a total of 23 compounds were upregulated, among which glycyrrhizin C2 was upregulated, while glycyrrhizin G2, apiosylglucosyl 4-hydroxybenzoate and 12 other compounds were downregulated (Figure 4E). In the mid-dry stage, there were a total of nine different compounds, which were significantly reduced compared to the previous stage, with five upregulating compounds and four downregulating compounds in epidermin, diacetone alcohol, and dihydronaringenin-O-sulphate (Figure 4F). From the mid-drying stage to the complete drying stage, there were a total of 15 different compounds, of which 10 were upregulated compounds and five were downregulated compounds (Figure 4G). The types of compounds changed greatly in the fresh period, and Figure 4E clearly shows that the content was relatively high. Related to the endobiotic species we obtained in the previous period, the types of compounds and endophytic microorganisms are the highest in the fresh period of licorice.

### Correlation Analysis Between Microorganisms and Host Metabolites During Drying

**Figure 5:**
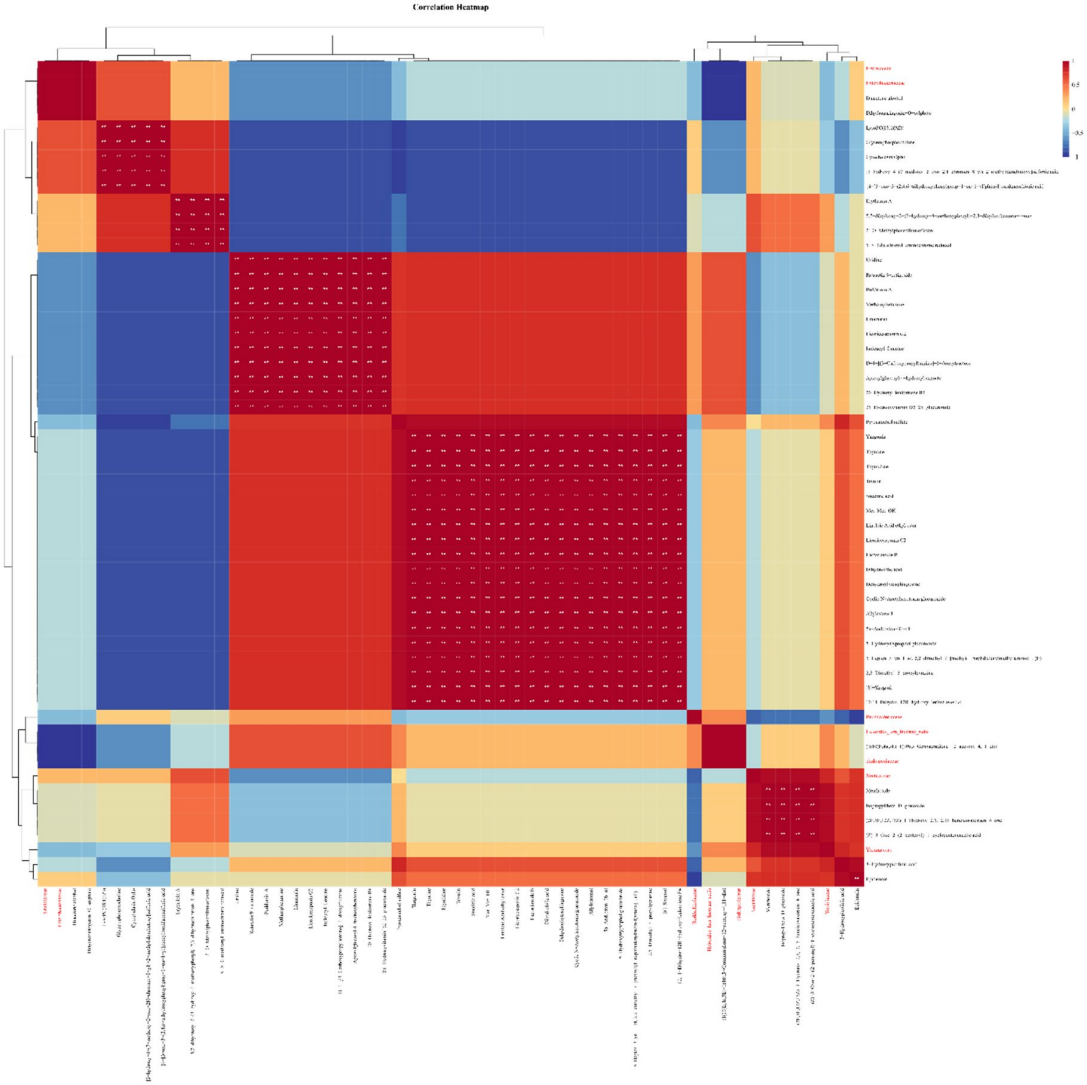
The correlation between the main metabolites and the main endophytic microorganisms of licorice (red font is the family of the microorganism, black font is the identified metabolite, the red square indicates positive correlation, and the blue square indicates negative correlation, ** is *p*<0.01).

Correlation analysis was carried out between the proportion of the dominant family of endophytic microorganisms and the differential metabolites of licorice during slow drying. The results showed that the endophytic fungi Cladosporiaceae and Helotiales_fam_Incertae_sedis and the endophytic bacteria Erwiniaceae and Enterobacteriaceae were significantly correlated with the metabolites diacetone alcohol and dihydronaringenin-O-sulphate (Figure 5). Yersiniaceae and Nectriaceae had a significant correlation with the metabolite menthoside (isoflavone glycoside).

## Discussion

### Fresh Processing Can Improve the Content of Active Ingredients in Licorice

The postharvest processing of Chinese medicinal materials is the most critical factor affecting their quality (Liu et al., 2019; Su et al., 2019). The biologically active ingredients of licorice dynamically changed during natural slow drying. We found that fresh licorice had a high content of liquiritin and glycyrrhizic acid, and the content of these components showed a downward trend with slow drying (Figure 1). This was consistent with most existing reports that the extension of the drying process time will adversely affect the content of active ingredients (Yabar et al., 2011). Therefore, fresh processing methods after harvesting licorice can reduce the loss of internal components and drying time, thus increasing drying efficiency. In addition, during the determination of the content of active ingredients, it was found that liquiritin, glycyrrhizic acid and other substances in the form of glycosides will slowly lose glucose molecules during the drying process to form aglycones. At present, many medicinal plants are processed when they are harvested fresh (Luz et al., 2009). With the advantage of fresh processing, an increasing number of producers will likely choose this method of fresh processing for medicinal material in the future.

### Diversity and Function of the Endophytic Microbial Community

According to previous studies, plants that can survive in extreme environments usually have a unique microbial community. These microbes play a very important role in improving host resistance and productivity (Habibzadeh et al., 2013; Coleman-Derr et al., 2016; Dai et al., 2020). We found that the families Nectriaceae and Enterobacteriaceae in completely dried licorice were the main families that existed after licorice drying, proving that they have a certain “drought resistance” property. These “drought-resistant” microorganisms may have a correlation with the content of active ingredients. For example, the content of isoliquiritigenin in this study had a very significant correlation with the abundance ratio of Cladosporiaceae, Helotiales-fam-incertae-sedis and Enterobacteriaceae (Supplementary Material 3). In addition, some studies have reported that the isolation of microorganisms from plants growing in arid environments can promote plant growth and drought resistance (Marasco et al., 2012; Fan et al., 2020; Dubey et al., 2021). It is of great significance to isolate and culture these “drought resistant” microorganisms to determine whether they can play a role in the host’s tolerance to natural drought stress and to further clarify whether they have an impact on the metabolic pathway of licorice. Therefore, we will continue to isolate and culture these “drought resistant” microorganisms in the future.

Endophytes may be involved in these pathways and affect the changes in the effective components of the host. For example, it has been reported that endophytic microorganisms are involved in the metabolic pathways of amino acids, causing differences in the metabolic profile of the host (Llorens et al., 2019). Some functional prediction analyses have shown that microorganisms have related enzymes and genes in the synthesis pathway of plant secondary metabolism. It has been speculated that these microorganisms have the ability to synthesize or transform secondary metabolites. In addition, we also found some beneficial enzymes in the prediction of microbial function, such as amylase and chitinase, which play an important role in degrading plant residues and plant polysaccharides (Kaewkla and Franco, 2021). The interaction between microorganisms and plants was also further demonstrated to some extent. PICRUSt2 can enrich our understanding of the diversity of internal microbial functions, as well as the proportion of these functions in the flora, and further provide support for the development and utilization of microorganisms. However, in view of the limitations of PICRUSt2, it will be further determined by combining metagenomics and transcriptomics. In addition, these endophytes can be further isolated and cultured to verify their functions.

### Endophytic Microorganisms May Cause Differences in Host Metabolites

We simulated the natural drying process of licorice and performed LC–MS metabolome analysis on licorice in the fresh, early and late drying stages. Licorice metabolites were quite different between the drying stages. The variety of different metabolites was the most abundant in the fresh period. At this time, the diversity of endophytic microorganisms in licorice was also the highest. It is speculated that the enzymes of the microorganisms in the fresh period of licorice have better activity and participate more in the metabolic pathway of the host. After drying for a period of time, the enzymes of the microorganisms or the host’s enzymes are gradually inactivated, which may be due to changes in various biochemical processes, including the participation of endophytic microorganisms in the formation of host metabolites and the action of enzymes or the activity of the host’s own enzymes. For example, it has been reported in the literature that *Salvia miltiorrhiza* has a rich microbial community, which helps the host plant’s genome encode metabolic processes, thus affecting the tanshinone synthesis pathway (Chen et al., 2018). This result further shows that endophytic microorganisms can indeed affect the metabolic pathway of the host, and the specific mechanism by which they interact needs further study. Subsequently, the correlation between the differential metabolites and the main endophytic microorganisms was analyzed, and the results showed that the endophytic microbial community has a certain correlation with the metabolites. Therefore, it can be inferred that host metabolites were also closely related to endophytic microorganisms. This may be caused by changes in host metabolites caused by endophytic microorganism metabolites or by more complex microbial-host interactions. However, how endogenous microorganisms affect host metabolites should continue to be studied further in the future.

## Conclusion

This study explored the dynamic changes in endophytic microorganisms, the content of active ingredients, and metabolites of licorice during natural drying. During the drying process, the decrease in water content caused the contents of glycyrrhizic acid and liquiritin to decrease, while the liquiritigenin and isoliquiritigenin contents increased. The microbial diversity in the roots of licorice also showed a downward trend of decreasing content. The endophytic fungus Cladosporiaceae and endophytic bacteria Burkholderiaceae were the dominant family when fresh, but Nectriaceae and Enterobacteriaceae were the dominant families after drying. In addition, the metabolomics results showed that the differential metabolites of licorice were reduced during the natural drying process. Correlation analysis between dominant families and differential metabolites showed that there was a certain correlation between the two. Based on these results, we suggest that fresh processing is better after harvest.

## Data Availability Statement

The datasets presented in this study can be found in online repositories. The names of the repository/repositories and accession number(s) can be found at: https://www.ncbi.nlm.nih.gov/, PRJNA748524.

## Author Contributions

TL, DJ, and CL designed the study and prepared the manuscript. GR participated in the experiments and data analysis. TL conducted the experiments and data analysis. DJ and CL revised the manuscript. All authors contributed to the article and approved the submitted version.

## Funding

This work gratefully acknowledges the financial support provided by the National Natural Science Foundation of China (81773838 and 81703645), 2019–2020 National Medical Products Administration Project Funding Project, and the Fundamental Research Funds for the Central Universities (Beijing University of Chinese Medicine, no. 2020-JYB-ZDGG-037).

## Conflict of Interest

The authors declare that the research was conducted in the absence of any commercial or financial relationships that could be construed as a potential conflict of interest.

## Publisher’s Note

All claims expressed in this article are solely those of the authors and do not necessarily represent those of their affiliated organizations, or those of the publisher, the editors and the reviewers. Any product that may be evaluated in this article, or claim that may be made by its manufacturer, is not guaranteed or endorsed by the publisher.
